# IFN Lambda Deficiency Contributes to Severe COVID-19 Outcomes

**DOI:** 10.3390/ijms251910530

**Published:** 2024-09-30

**Authors:** Anna Zaleska, Anna Dor-Wojnarowska, Anna Radlińska, Marta Rorat, Wojciech Szymański, Adrian Gajewski, Maciej Chałubiński

**Affiliations:** 1 Department of Allergology and Internal Medicine, Wroclaw Medical University, 50-368 Wroclaw, Poland; anna.dor-wojnarowska@umw.edu.pl (A.D.-W.); anna.radlinska@umw.edu.pl (A.R.); 2 Department of Social Sciences and Infectious Diseases, Wroclaw University of Science and Technology, 50-370 Wroclaw, Poland; marta.rorat@pwr.edu.pl; 3 Department of Infectious Diseases and Hepatology, Wroclaw Medical University, 50-367 Wroclaw, Poland; 4 Department of Immunology and Allergy, Medical University of Lodz, 92-213 Lodz, Poland; adrian.gajewski@umed.lodz.pl (A.G.); maciej.chalubinski@umed.lodz.pl (M.C.)

**Keywords:** interferon, innate immunity, SARS-CoV-2, COVID-19

## Abstract

Interferons (IFNs) produced by airway epithelial cells are crucial in defending against pathogens. Fluctuations in IFN-λ levels may influence coronavirus disease 19 (COVID-19) severity. However, conflicting data have been reported regarding serum IFN-λ concentrations in COVID-19 patients. To address this, we evaluated serum IFN-λ levels over time in moderate and severe COVID-19 patients and their association with cytokine production and clinical parameters using the enzyme-linked immunosorbent assay (ELISA) and the Bio-Plex Pro Human Cytokine 17-plex Assay. Results from testing 51 COVID-19 patients showed that 68% lacked detectable serum IFN-λ. Among non-IFN-λ secretors, severe COVID-19 predominated. In contrast, IFN-λ secretors displayed stable IFN-λ levels in moderate cases, while severe cases showed a decline over time, which persisted even after recovery. A negative correlation was observed between IFN-λ levels and inflammatory markers. This, combined with an increase in tumor necrosis factor alpha (TNF-α) and clinical improvement, suggests a regulatory role for IFN-λ in promoting faster recovery. Despite this, survival rates were similar between the groups, indicating that while IFN-λ influences the course of the disease, it does not directly affect overall survival. In conclusion, IFN-λ is vital, but not unique, for the antiviral response and COVID-19 recovery. Simultaneously, serum IFN-λ deficiency signifies severe COVID-19.

## 1. Introduction

Coronavirus disease 19 (COVID-19), with a high mortality rate, has been an important healthcare problem in recent years. Despite mass vaccinations that began at the end of 2020, which forced virus mutations and contributed to a milder course of the disease, severe cases of COVID-19 still occur, posing a challenge for clinicians and putting patients at risk of health complications and life-threatening outcomes [1]. Many factors contributing to the severity of COVID-19 have already been recognized, including age, ethnicity, gender, immunodeficiency, vaccination status, and comorbidities, such as diabetes, hypertension, and obesity [2]. However, our understanding of immunological markers that can identify patients predisposed to severe COVID-19 remains insufficient. Some data point to persistent eosinopenia, lymphopenia, and elevated interleukin (IL)-6 level as indicators of severe disease [3]. Interferons (IFNs), particularly type I and III, which are part of the innate immune system, act to limit viral replication and assembly [4]. In response to viral infection, IFNs stimulate downstream signaling through IFN receptors (type I IFNs through IFNAR1 and/or IFNAR2, and type III IFNs through IFNλR1 and/or interleukin (IL)10Rβ), which induces the expression of IFN-stimulated genes (ISGs) and the production of proteins with various antiviral functions [5,6]. Proper (dose-dependent) IFN action in the early stage of COVID-19 may lead to effective viral clearance and an asymptomatic/mild course of the disease. In mammalian epithelial cell lines, type I and III interferons inhibited severe acute respiratory syndrome coronavirus 2 (SARS-CoV-2), and initial high levels of IFNs were correlated with better outcomes [7]. However, SARS-CoV-2 has evolved evasive strategies to escape host control. Open reading frames (ORFs), papain-like protease (PLpro), and nonstructural proteins (NSP1, NSP14) produced by SARS-CoV-2 can disrupt the IFN signaling pathway, leading to the impaired induction and function of IFNs [4]. Inherent defects in the host’s immune system and the presence of neutralizing autoantibodies against IFNs also affect the activity and levels of IFNs in plasma [8]. This is reflected by the final outcome of the IFN response, where a deficient or delayed response promotes hyper-inflammation and lung injury [9]. Type III IFNs-λ of interest include IFN-λ1, IFN-λ2, IFN-λ3, and IFN-λ4, and exhibit similar antiviral activity to type I IFN-α but are devoid of inflammatory effects. This disparity grants IFN-λ an advantage over IFN-α, as an increase in IFN-λ levels, whether endogenous or exogenous, during the later stage of COVID-19 does not trigger hyper-inflammation (known as a cytokine storm) and multi-organ damage associated with IFN-α. Type III interferons (PEGylated IFN-λ1a) have been tested in several clinical trials, with promising results for both preventing and treating COVID-19 [10,11,12]. However, the pathophysiological role of IFN-λ, which may impair the epithelial barrier and predisposes to bacterial superinfection, remains controversial [13,14]. Excessive or prolonged IFN-λ production has been found to induce neutrophil chemotaxis and activate the complement/coagulation cascade, exacerbating the disease [15]. Attention was also drawn to the usefulness of IFN-λ in diagnostics. In Japan, serum interferon lambda 3 (IFN-λ3) has been commercially used to asses COVID-19 severity and progression to severe cases [16,17]. Nonetheless, research data have been contradictory and inconclusive. Most studies have reported a reduction or absence of IFN-λ in the serum of patients with severe COVID-19 [18,19,20], while some data suggest the opposite [16,21]. Additionally, the optimal timing for measuring serum IFN-λ as an indicator of COVID-19 exacerbation is still pending. The limited data encourage further exploration of this subject. In this context, we aimed to assess changes in serum IFN-λ levels over time in COVID-19 patients and their association with cytokine production, clinical parameters, and disease severity. Particular attention was paid to differences between patients with complete serum IFN-λ deficiency and those who are IFN-λ secretors.

## 2. Results

### 2.1. Clinical Data and Disease Severity

There were no significant differences between non-IFN-λ-secretors (*n* = 35) and IFN-λ-secretors (*n* = 16) with regard to age, gender, body mass index (BMI), pulmonary infiltration area, and applied treatment (Table 1). In both groups, the prevalent symptoms of COVID-19 were fever, dry cough, and dyspnea, with only sore throat being significantly more frequent (*p* = 0.025) in patients secreting IFN-λ (Table 1). No significant differences were observed in terms of comorbidities between the groups of non-IFN-λ-secretors and IFN-λ-secretors, with hypertension and hypothyroidism being the most common (Table 1). In the group of individuals who did not produce IFN-λ, 57% of patients experienced severe COVID-19, while 31% had moderate COVID-19. In contrast, among those who secreted IFN-λ, the rates of severe and moderate COVID-19 were both 38%. The distinction in disease severity between the IFN-λ-secreting and non-secreting individuals did not show significant variation. Critical cases of COVID-19 were observed in both sets of patients, including those not producing IFN-λ (11%) and those secreting it (25%), numbering four patients in each group (Table 1).

### 2.2. IFN-λ

**Figure 1:**
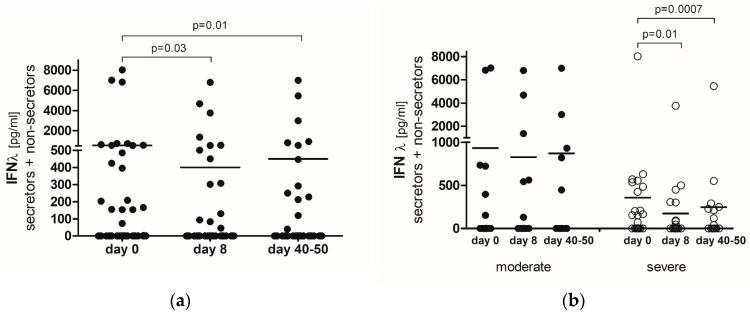
(**a**) IFN-λ concentration in the serum of COVID-19 patients (*n* = 51) at three time points. (**b**) IFN-λ concentration in serum of COVID-19 patients (*n* = 51) regarding disease severity. Results are presented as row data with the average concentration for each group (*p*—statistical significance). (day 0) the first day of hospitalization, (day 8) the 8th day of hospitalization, (day 40–50) after hospital release between days 40 and 50.

Type III IFN-λ was not detected in the serum of 35 (68%) out of 51 COVID-19 patients tested. A significant decrease in IFN-λ levels was observed on day 8 (*p* = 0.03) and between days 40 and 50 (*p* = 0.01) in comparison with day 0 (the first day of hospitalization) (Figure 1a).

An analysis of the relationship between the IFN-λ concentration and disease severity showed that average IFN-λ levels were higher in moderate COVID-19 patients than in severe ones at all time points, though these differences were not statistically significant (Figure 1b). Additionally, severe COVID-19 patients exhibited a significant decline in IFN-λ levels on day 8 (*p* = 0.01) and between days 40 and 50 (*p* = 0.0007) (Figure 1b).

Furthermore, on day 8, a positive correlation was identified between IFN-λ and both gamma-glutamyl transpeptidase (GGT) (r = 0.4; *p* = 0.02) and D-dimer (r = 0.3; *p* = 0.04). In both cases, a decrease in IFN-λ levels coincided with a reduction in GGT and D-dimer concentrations (Table 2). In parallel, IFN-λ was inversely related to procalcitionin (r = −0.4; *p* = 0.008) and fibrinogen (r = −0.4; *p* = 0.009) (Table 2). No correlation was found between IFN-λ levels and the percentage of lung involvement due to COVID-19 (r = 0.15; *p* = 0.27) on the day of hospital admission (day 0) (Figure S1). 

### 2.3. IFN-λ-Secretors

**Figure 2:**
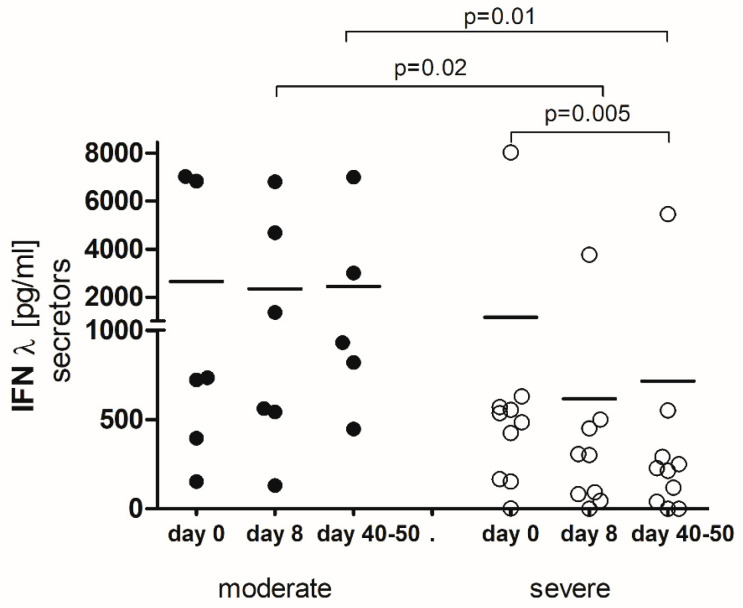
Serum IFN-λ concentration in IFN-λ-secreting individuals with COVID-19 (*n* = 16) by disease severity. Results are presented as row data with the average concentration for each group (*p*—statistical significance). (day 0) the first day of hospitalization, (day 8) the 8th day of hospitalization, (day 40–50) after hospital release between days 40 and 50.

Focusing specifically on IFN-λ-secretors (*n* = 16) revealed relevant differences between moderate and severe COVID-19 patients. Moderate cases displayed significantly higher levels of IFN-λ compared to severe ones on day 8 (*p* = 0.02) and between days 40 and 50 (*p* = 0.01) (Figure 2). Moreover, a notable decrease in IFN-λ was noted in the severe group between days 40 and 50 (*p* = 0.005) (Figure 2). 

### 2.4. Cytokine Analysis

Out of the 17 cytokines tested, nine (IL-1β, IL-2, IL-4, IL-5, IL-12, IL-13, IL-17, granulocyte colony-stimulating factor (G-CSF), granulocyte-macrophage colony-stimulating factor (GM-CSF)) were undetectable in the peripheral blood of COVID-19 patients. 

**Figure 3:**
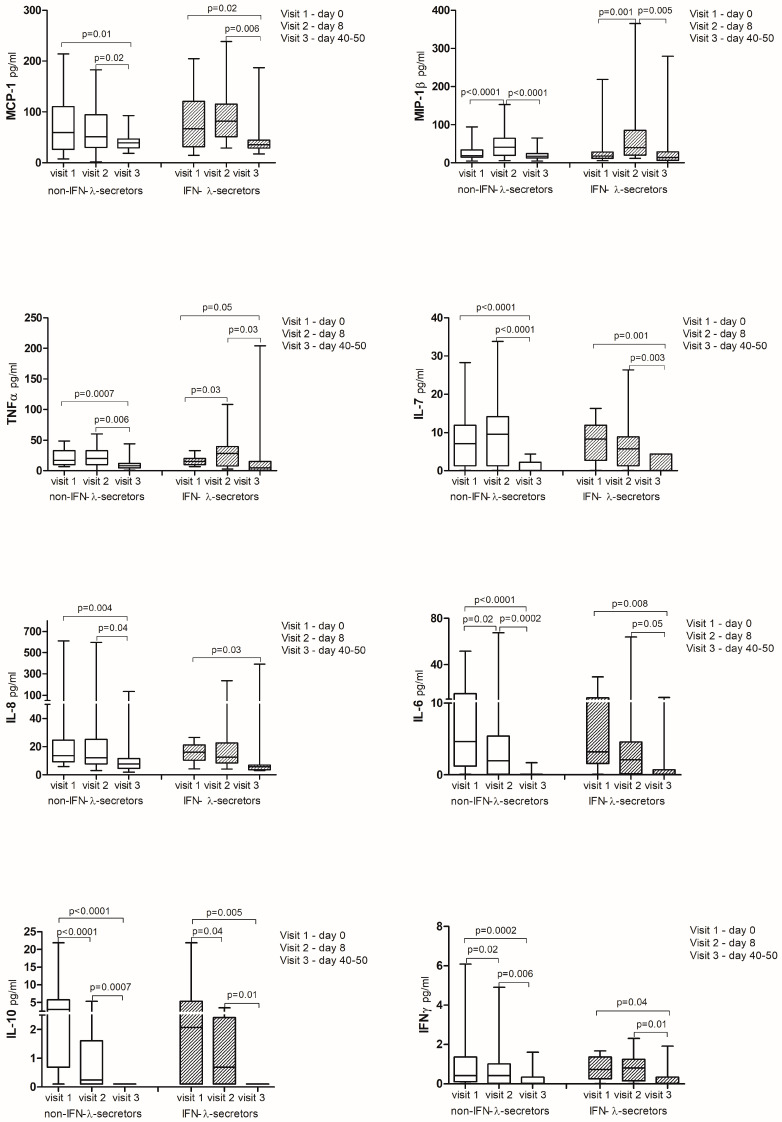

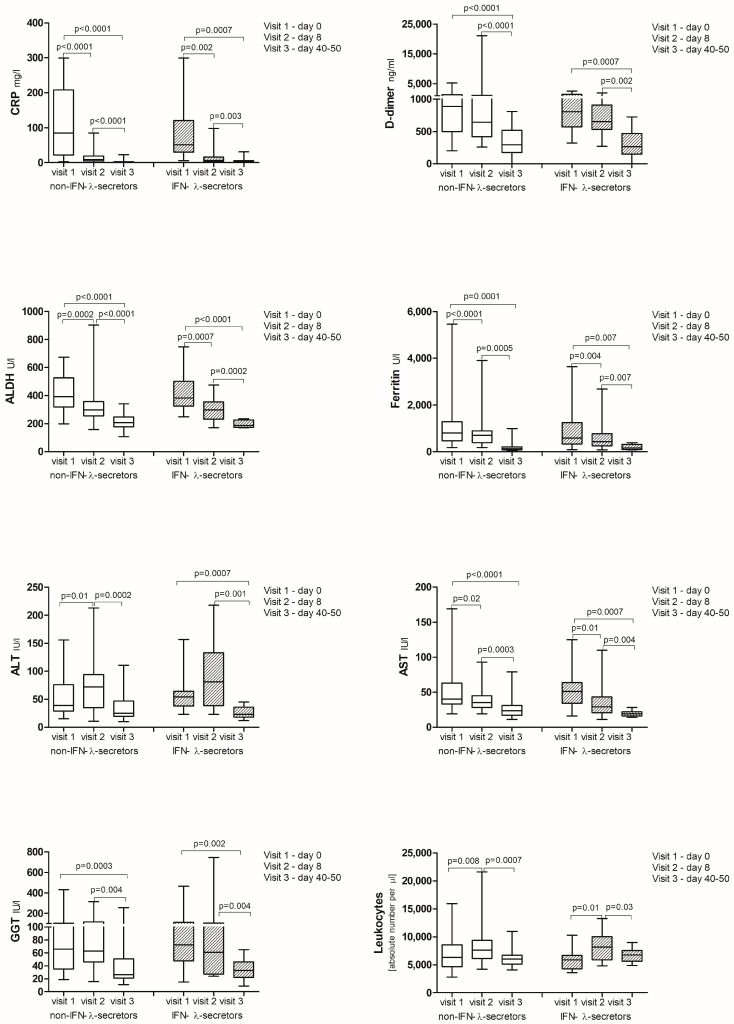

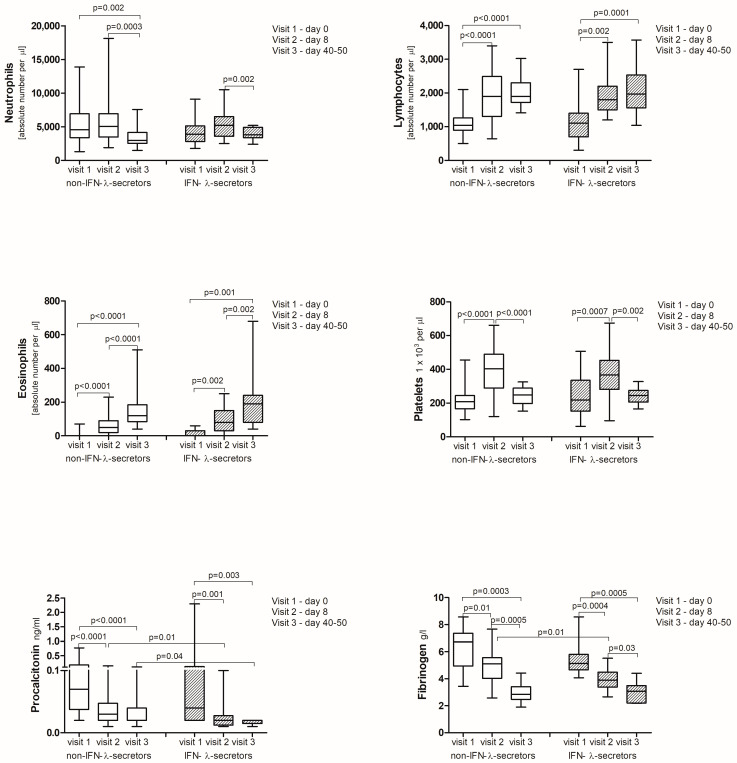
Cytokine repertoire in IFN-λ-secreting (*n* = 16) and non-secreting individuals (*n* = 35) with COVID-19 at three time points. Results are presented as row data with medians for each group (*p*—statistical significance).

There were no significant differences in the levels of monocyte chemoattractant protein-1 (MCP-1), macrophage inflammatory protein-1β (MIP-1β), tumor necrosis factor α (TNF-α), IL-7, IL-8, IL-6, IL-10, and IFN-γ between non-IFN-λ-secretors and IFN-λ-secretors at any time point studied (Figure 3). However, higher concentrations of procalcitonin on day 8 (*p* = 0.01) and between days 40 and 50 (*p* = 0.04), as well as fibrinogen on day 8 (*p* = 0.01), were observed in non-IFN-λ-secretors (Figure 3).

Regardless of IFN-λ secretion, a significant increase in the levels of MCP-1, TNF-α, IL-8, IL-6, IL-10, and IFN-γ, as well as clinical parameters, including c-reactive protein (CRP), D-dimer, aldehyde dehydrogenase (ALDH), ferritin, procalcitonin, fibrinogen, and aspartate aminotransferase (AST), was noted on the first day of hospitalization (day 0) in all patients with a simultaneous significant decrease in the levels of lymphocytes, eosinophils, and platelets. Eight days later (day 8), further significant growth was found in the levels of MCP-1, TNF-α, and IFN-γ, accompanied by newly elevated MIP-1β, leukocytes, neutrophils, and alanine aminotransferase (ALT), which all finally dropped between days 40 and 50. Additionally, on day 8, opposite to day 0, there was an elevation of lymphocytes, eosinophils, and platelets, along with a reduction in IL-8, IL-6, IL-10, procalcitonin, and fibrinogen (Figure 3—all corresponding *p* values are provided in the graphs). 

## 3. Discussion

IFN-λ with antiviral activity has been shown to be protective against viral infections, such as influenza, and exert longer supportive effects than IFN-α [22]. A similar effect of IFN-λ has been found in the case of SARS-CoV-2 [7]. Interestingly, increased serum IFN-λ levels were present mostly in non-severe cases of COVID-19 [18,19,20,23]. Additionally, Blanco-Melo et al. [24], using a variety of model systems, showed that the host response to SARS-CoV-2, unlike other respiratory viruses, is unable to activate a robust IFN-I and III response. Despite a high viral load and replication, there was no activation of TANK-binding kinase 1 (TBK1), which is responsible for IFN-I and IFN-III expression, nor any induction of signal transducer activator of transcription 1 (STAT1) or myxovirus resistance 1 (MX1). IFN-I and III gene expression was undetectable in lung biopsies via RNA sequencing or semiquantitative polymerase chain reaction (PCR), and serum samples from COVID-19 patients were free of IFN-β and IFN-λ [24]. Thus, a deficiency or lack of serum IFN-λ is perceived as an unfavorable sign in the course of COVID-19. In our study, critical or severe cases of COVID-19 were observed in both groups of individuals: those with a complete lack of serum IFN-λ and those with elevated levels. However, a higher percentage of severe cases were associated with the absence of serum IFN-λ. Patients with detectable IFN-λ had higher levels during moderate cases, which remained elevated throughout the disease course and recovery (at 40–50 days), potentially contributing to a lower disease activity. This aligns with a previous finding that IFN-λ inhibits SARS-CoV-2 replication in a dose-dependent manner [7,25]. Interestingly, Fukuda et al. [19] reported that serum IFN-λ2 and IFN-λ3 levels decreased until becoming PCR-negative, though they did not distinguish between severe and mild COVID-19 patients [19]. In contrast, our study found that only severe cases experienced a decline in IFN-λ over time, which remained low after recovery. This suggests a possible defect in the innate immunity of severe patients or the resolution of inflammation. However, survival rates were similar in both IFN-λ secretors and non-IFN-λ secretors, with all patients surviving except for one. This indicates that other factors, rather than IFN-λ alone, may play a significant role in determining survival in COVID-19. On the other hand, identifying patients with low or undetectable serum IFN-λ at the onset of the disease may help predict its course. This is supported by the findings of Lucas et al. [26] and Fukuda et al. [19], who confirmed this through multivariate analysis. This also renders IFN-λ a potentially valuable therapeutic tool against COVID-19. 

Nevertheless, patients with critical/severe COVID-19 and high levels of IFN-λ induce curiosity. In Japan, where serum IFN-λ3 has been commercially used to asses COVID-19 progression, Sekimoto et al. [16] showed that patients negative for both IFN-λ3 and chemokine (C-C motif) ligand 17 (CCL17) did not experience disease progression, whereas elevated IFN-λ levels heralded the advancement of COVID-19 [16]. In our severe COVID-19 patients, although the initial serum IFN-λ levels were elevated, they diminished in subsequent days. Since the severe condition typically develops on days 5–6 following SARS-CoV-2 infection, the observed decrease in serum IFN-λ on day 8 may have been linked to disease exacerbation. The negative correlation between IFN-λ levels and inflammatory markers, like procalcitonin and fibrinogen, at this time may support this assertion. This is also in line with the results of Suzuki et al. [17] who showed that IFN-λ surged at the beginning of infection and then dropped suddenly before the development of severe disease (on days 5–7), requiring oxygen support. However, we have to take into consideration that the 8th day following hospital admission for many of our patients was actually the 12th or 13th day following SARS-CoV-2 infection, because patients were admitted with developed symptoms of pneumonia and dyspnea. Therefore, the decline in IFN-λ on day 8 could indicate the resolution of inflammation and recovery rather than exacerbation, especially given the average hospitalization time of 11 days. In turn, although inflammatory markers remained elevated on day 8, they were lower than on the day of admission and continued to decrease. Taken together, we might hypothesize that the increased IFN-λ at admission represents a delayed production, usually seen on days 5–6 post-infection, coinciding with the rise in IFN-α and signaling exacerbation [27].

Remarkably, not all studies have linked IFN-λ levels with disease severity. Tovo et al. [28] observed sustained serum IFN-λ expression in children regardless of COVID-19 severity, while Kim et al. [21] found similar plasma levels of IFN-λ1/3 in hospitalized patients with non-severe COVID-19 and those in the intensive care unit (ICU). Although the first findings can be explained by an age-associated impairment in the phosphorylation of interferon regulatory factor 7 (IRF-7) and a reduced function of plasmacytoid dendritic cells in older adults, which are responsible for controlling viral infection through the release of IFN-λ [29], the second suggests that the severe course of COVID-19 may be influenced by other agents. Notably, in our study, a few moderate COVID-19 cases lacked serum IFN-λ, which did not worsen the disease.

Despite low innate antiviral defense, as evidenced by reduced or absent serum IFN-λ, SARS-CoV-2 triggered a strong chemotactic and inflammatory response. A quantitative analysis of cytokines showed a general increase in the first days of infection without significant differences between IFN-λ-secretors and non-IFN-λ-secretors. MCP-1, TNF-α, IL-8, IL-6, IL-10, IFN-γ, and inflammatory parameters were elevated on day 0 in all patients, with a simultaneous significant decrease in the levels of lymphocytes, eosinophils, and platelets, consistent with the findings of other authors [30,31,32]. The marked changes involved inflammatory indicators, such as procalcitonin and fibrinogen, both of which were significantly higher on admission and 8 days later in the group of individuals who did not produce IFN-λ, suggesting a more severe course of the disease in these patients. Interestingly, TNF-α levels increased significantly on day 8 in IFN-λ-secretors. As TNF-α, while proinflammatory, also exhibits a tolerizing effect by desensitizing monocytes and macrophages to additional Toll-like receptor (TLR) stimulation, this likely helped limit excessive inflammation and promote faster COVID-19 resolution [33]. The observed decrease in CRP, IL-6, procalcitonin, fibrinogen, D-dimer, ALDH, and ferritin, along with increases in eosinophils and lymphocytes at that time, as well as an improvement in patients’ conditions, confirm this thesis. 

The pathophysiological role of IFN-λ, as discussed by Broggi et al. [13] and Read et al. [15], is also noteworthy. While IFN-λ is linked to viral clearance, it can also, in certain circumstances (such as chronic or exaggerated production), contribute to inflammation and fibrosis in viral and non-viral diseases affecting the liver, lungs, and skin. Despite different disease etiologies, disruption of the epithelial barrier and/or exacerbation by microbial infection triggering immune pathway activation are common [15]. This ultimately drives inflammation and tissue damage, worsening disease progression. A similar mechanism involving impaired barrier function is seen in atopic dermatitis, though in this case, it is connected with filaggrin mutations [34]. Regardless, the greatest threat remains superinfection. In our study, we generally did not observe secondary bacterial infections, except in one patient who died from sepsis. D-dimer levels, related to coagulation activity, and neutrophil counts showed no significant differences between IFN-λ-secretors and non-IFN-λ-secretors. The observed rise in neutrophils on day 8 was attributed to corticosteroid treatment. Simultaneously, the stable and long-lasting production of IFN-λ was associated with moderate COVID-19. 

This study exhibited several limitations. Firstly, a limited number of patients were included, which likely influenced the significance of the analysis. Secondly, there was no healthy group and patients with mild disease severity. 

## 4. Materials and Methods

### 4.1. Examined Group and Biochemical Analysis

The study included 51 hospitalized patients, 36 males and 15 females, aged from 31 to 72 years (average age 53.3 ± 11.5 standard deviation, SD) with moderate (average mean 54.8 ± 10.8 SD) to severe (average age 52.4 ± 12.1 SD) COVID-19. The diagnosis of COVID-19 was made on the basis of clinical symptoms and a positive polymerase chain reaction (PCR) test from a nasopharyngeal swab on the day of admission. Disease severity was determined based on the National Institute of Health COVID-19 treatment guidelines [35]. According to this classification, COVID-19 patients were categorized as moderate (*n* = 17) if they showed evidence of lower respiratory disease (pneumonia) or imaging and who had an oxygen saturation (SpO_2_) ≥ 94% on room air at sea level and severe (*n* = 34) if they had SpO_2_ ≤ 94% on room air at sea level, a ratio of arterial pressure of oxygen to fraction of inspired oxygen (PaO_2_/FiO_2_) < 300 mmHg, a respiratory rate ≥ 30 breaths/min, or lung infiltrates > 50%. In the severe group, patients with a critical course of COVID-19 who presented with respiratory failure, septic shock, and/or multiple organ dysfunction were additionally distinguished (*n* = 8). The average hospitalization time was 10.8 ± 3.8 (SD) days. One patient died. The serum concentrations of IFN-λ were measured using the specific enzyme-linked immunosorbent assay (theHuman IL-28A/IFN-lambda2 - DuoSet ELISA) kits (R&D Systems, Minneapolis, MN, USA), following manufacturers’ instructions. The Bio-Plex Pro Human Cytokine 17-plex assay (Biorad, Hercules, CA, USA) was used for the quantitative evaluation cytokines and chemokines in serum. All measurements were performed at three checkpoints: on day 0 (the first day of hospitalization), on day 8, and between days 40 and 50. Serum samples were taken from each subject and stored at −80 °C until use. Based on the results of serum IFN-λ concentrations, patients were divided into two groups: IFN-λ-secretors (*n* = 16) and non-IFN-λ-secretors (*n* = 35) and further analyzed. The comparative analysis was performed in relation to clinical data that were collected and analyzed retrospectively. Laboratory parameters, including a complete blood count (CBC), C-reactive protein (CRP), fibrinogen, procalcitonin, D-dimer, aldehyde dehydrogenase (ALDH), gamma-glutamyl transferase (GGT), aspartate aminotransferase (AST), alanine aminotransferase (ALT), and ferritin in sera, were routinely performed in the hospital diagnostic unit using applicable diagnostic methods. Detailed characteristics of patients are presented in (Table 1).

### 4.2. Computed Tomography

Chest computed tomography (CT) was performed on the first day of hospitalization, using a 128-slice multidetector CT scanner, employing automatic exposure control for radiation dose reduction and optimization. Patients were examined with a dedicated non-contrast chest scanning protocol, in the supine position, and in the craniocaudal direction. CT scans were made with full inspiration and breath-holding. Spatial images were obtained through multiplanar reconstructions. The analysis focused on identifying and assessing ground-glass opacities, lung consolidations, reticular and streak opacities, vascular dilatations, nodules, and other relevant features. The results were presented in a descriptive manner, including the percentage of the lung involved. 

### 4.3. Statistical Analysis

Statistical analyses were performed using GraphPad Prism 5 (GraphPad Software, Inc., La Jolla, CA, USA). In the analysis of continuous variables, non-parametric tests were used: Wilcoxon Signed Rank test, Mann–Withney U test, and Kruskal–Wallis test with Dunn’s Multiple Comparison. Categorical variables were compared using the Fisher’s exact test. In general, data are expressed as medians. In several cases that required it, data were expressed as means. A *p* value < 0.05 was considered significant. For correlation analyses, the Spearman correlation test was used.

## 5. Conclusions

Serum IFN-λ level should be considered in assessing the COVID-19 progression risk. Special attention should be paid to the patient’s condition to accurately determine disease severity. The time elapsed since disease onset must be carefully estimated to correctly interpret IFN-λ results. Low or undetectable serum IFN-λ should be seen as indicator of severe disease. Repeated IFN-λ measurements are recommended, and in the event of a delayed increase, the potential for an impending exacerbation should be considered, along with modification of the current treatment. 

We believe that our findings contribute to the understanding of COVID-19 pathophysiology. However, further research involving a larger cohort of patients than those studied here is necessary to confirm the utility of IFN-λ measurements in practice. 

## Figures and Tables

**Table 1:** Clinical characteristics of COVID-19 patients.

	IFN-λ-Secretors	Non-IFN-λ-Secretors	*p*
	(*n* = 16)	(*n* = 35)	
Age, mean ± SD	51.6 ± 11.3	54.1 ± 11.7	*p* = 0.434
Female, *n*	6 (38%)	9 (26%)	*p* = 0.510
Male, *n*	10 (62%)	26 (74%)	*p* = 0.510
BMI kg/m^2^, mean ± SD	30.7 ± 5.1	29.0 ± 4.0	*p* = 0.272
% lung infiltrates, mean ± SD	55.9 ± 21.5	54.3 ± 22.6	*p* = 0.775
Symptoms, *n*:			
- fever	16 (100%)	34 (97%)	*p* = 0.161
- dry cough	13(81%)	33 (94%)	*p* =0.309
- dyspnea	15 (94%)	32 (91%)	*p* = 1.000
- muscle pain	7 (44%)	17 (49%)	*p* = 0.772
- headache	5 (31%)	14 (40%)	*p* = 0.756
- diarrhea	6 (38%)	10 (29%)	*p* = 0.534
- loss of taste	5 (31%)	12 (34%)	*p* = 1.000
- loss of smell	6 (38%)	9 (26%)	*p* =0.510
- weakness	0	5 (14%)	*p* = 0.167
- sore throat	5 (31%)	2 (6%)	*p* = 0.025 *
- nausea	2 (13%)	4 (11%)	*p* = 1.000
- vomiting	1 (6%)	2 (6%)	*p* = 1.000
- rash	1 (6%)	1 (3%)	*p* = 0.533
- sleep disturbance	0	2 (6%)	*p* = 1.000
- bronchospasm	0	1 (3%)	*p* = 1.000
Comorbidities, *n*:			
- hypertension	3 (19%)	16 (46%)	*p* = 0.117
- diabetes	2 (13%)	4 (11%)	*p* = 1.000
- hypothyroidism	1 (6%)	5 (14%)	*p* = 0.651
- asthma	3 (19%)	3 (9%)	*p* = 0.362
- allergic rhinitis	1 (6%)	4 (11%)	*p* = 1.000
- food allergy	1 (6%)	0	*p* = 0.313
- ischemic heart disease	1 (6%)	3 (9%)	*p* = 1.000
- gout	0	5 (14%)	*p* = 0.167
- psoriasis	2 (13%)	1 (3%)	*p* = 0.228
- arrhythmia	2 (13%)	1 (3%)	*p* = 0.228
- prostate hyperplasia	2 (13%)	1 (3%)	*p* = 0.228
- varicose disease	1 (6%)	1 (3%)	*p* = 0.533
- nephrolithiasis	1 (6%)	1 (3%)	*p* = 0.533
- reflux disease	1 (6%)	1 (3%)	*p* = 0.533
- heart failure	0	1 (3%)	*p* = 1.000
- sleep apnea	0	1 (3%)	*p* = 1.000
- chronic sinusitis	0	1 (3%)	*p* = 1.000
- COPD	0	1 (3%)	*p* = 1.000
- chronic kidney disease	0	1 (3%)	*p* = 1.000
- adrenal adenoma	0	1 (3%)	*p* = 1.000
- osteoporosis	1 (6%)	0	*p* = 0.313
Disease severity:			
Moderate COVID-19, n	6 (38%)	11 (31%)	*p* = 0.753
Severe COVID-19, *n*	6 (38%)	20 (57%)	*p* = 0.236
Critical COVID-19, *n*	4 (25%)	4 (11%)	*p* = 0.239
Treatment:			
Systemic GKS, n	10 (62%)	26 (74%)	*p* = 0.510
Remdesivir, *n*	7 (43%)	17 (48%)	*p* = 0.772
Tocilizumab, *n*	1 (6%)	1 (3%)	*p* = 0.533
Oxygen, *n*	15 (94%)	34 (97%)	*p* = 0.533

Abbreviations: *n*—number of patients; *p*—statistical significance; * statistically significant result; SD—standard deviation; COPD—chronic obstructive pulmonary disease; GKS—glucocorticoids; BMI—body mass index.

**Table 2:** Correlation between serum concentrations of IFN-λ and immunological parameters in COVID-19 patients (*n* = 51).

	Day 0	IFN-λ		Day 8	IFN-λ		Δ	IFN-λ	
		r	*p*		r	*p*		r	*p*
MCP-1	V1	0.17	ns	V2	0.16	ns	V2 − V1	0.12	ns
MIP-1β	V1	0.01	ns	V2	−0.03	ns	V2 − V1	−0.15	ns
TNF-α	V1	−0.11	ns	V2	−0.01	ns	V2 − V1	−0.13	ns
IL-7	V1	0.15	ns	V2	−0.14	ns	V2 − V1	0.26	ns
IL-8	V1	0.08	ns	V2	−0.27	ns	V2 − V1	0.11	ns
IL-6	V1	−0.01	ns	V2	−0.08	ns	V2 − V1	0.16	ns
IL-10	V1	−0.15	ns	V2	−0.05	ns	V2 − V1	0.04	ns
IFN-γ	V1	0.15	ns	V2	0.04	ns	V2 − V1	0.02	ns
CRP	V1	−0.03	ns	V2	−0.21	ns	V2 − V1	0.11	ns
ALT	V1	0.14	ns	V2	0.06	ns	V2 − V1	−0.19	ns
AST	V1	0.08	ns	V2	−0.25	ns	V2 − V1	−0.02	ns
GGT	V1	0.11	ns	V2	−0.18	ns	V2 − V1	0.44 *	0.02
D-dimer	V1	0.21	ns	V2	−0.05	ns	V2 − V1	0.29 *	0.04
ALDH	V1	0.01	ns	V2	−0.15	ns	V2 − V1	0.23	ns
Ferritin	V1	−0.03	ns	V2	−0.26	ns	V2 − V1	0.21	ns
Leukocytes	V1	−0.04	ns	V2	0.02	ns	V2 − V1	0.03	ns
Lymphocytes	V1	−0.01	ns	V2	0.08	ns	V2 − V1	0.16	ns
Neutrophils	V1	−0.08	ns	V2	−0.04	ns	V2 − V1	0.01	ns
Eosinophils	V1	0.25	ns	V2	0.25	ns	V2 − V1	0.01	ns
Platelets	V1	0.06	ns	V2	−0.07	ns	V2 − V1	0.22	ns
Procalcitonin	V1	−0.10	ns	V2	−0.38 *	0.008	V2 − V1	0.20	ns
Fibrinogen	V1	−0.06	ns	V2	−0.45 *	0.009	V2 − V1	−0.07	ns

V1 (day 0)—first day of hospitalization; V2 (day 8)—eight day of hospitalization; Δ (V2 − V1)—the values for V2 minus V1; r—correlation coefficient; *p*—statistical significance; * statistically significant result; ns—non-significant result. MCP-1—monocyte chemoattractant protein-1; MIP-1β—macrophage inflammatory protein-1 beta; TNF-α—tumor necrosis factor alpha; IL—interleukin, IFN—interferon; CRP—C-reactive protein; ALT—alanine aminotransferase, AST—aspartate aminotransferase; GGT—gamma-glutamyl transpeptidase; ALDH—aldehyde dehydrogenase.

## Data Availability

The data that support the findings of this study are available from the corresponding author upon request.

## Supplementary Materials

The following supporting information can be downloaded at: https://www.mdpi.com/article/10.3390/ijms251910530/s1.
