# Supplementary material for: IFN Lambda Deficiency Contributes to Severe COVID-19 Outcomes
Source: Int J Mol Sci. 2024 Sep 30;25(19):10530. doi: 10.3390/ijms251910530 (PMC11476353; doi:10.3390/ijms251910530)
Supplement: Supplementary file 1 [file ijms-25-10530-s001.zip › ijms-3190840-supplementary.pdf]

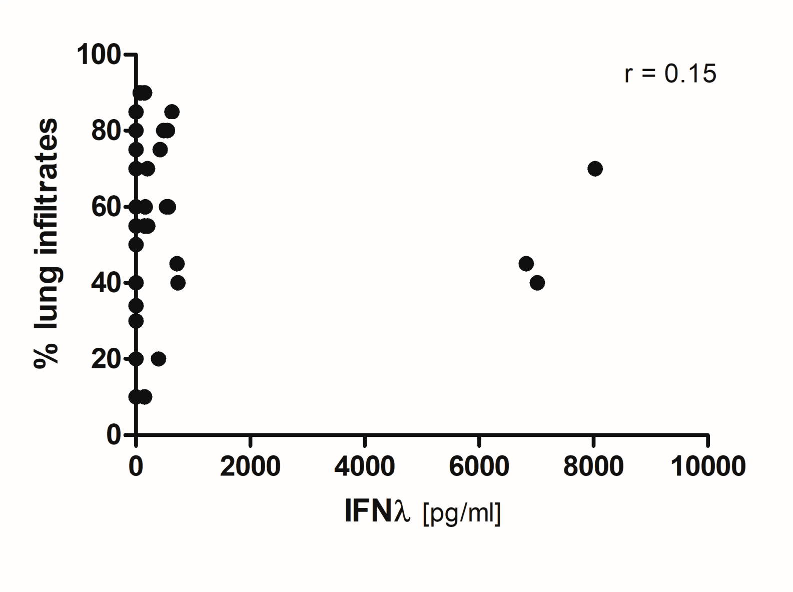

Figure S1. Correlation between serum IFN-λ level and % lung infiltrates in COVID-19 patients (n = 51) on the day of hospital admission (day 0).
